# Mentorship across institutional contexts: Insights from biology and biomedical science undergraduates at two institutions

**DOI:** 10.1371/journal.pone.0355290

**Published:** 2026-08-19

**Authors:** Héctor G. Loyola Irizarry, Krista Donis, Roxana González, Mia Uzcategui, Melissa McCartney

**Affiliations:** 1 Department of Natural Sciences, Caldwell University, Caldwell, New Jersey, United States of America; 2 Department of Biology Teaching and Learning, University of Minnesota Twin Cities, Minneapolis, Minnesota, United States of America; 3 Department of Biological Sciences, University at Buffalo, Buffalo, New York, United States of America; 4 Department of Biological Sciences, Florida International University, Miami, Florida, United States of America; 5 Department of Pharmacology and Toxicology, University at Buffalo, Buffalo, New York, United States of America; Lamar University, UNITED STATES OF AMERICA

## Abstract

Mentoring is essential for the development of biology and biomedical science (BBS) students, providing multiple benefits, including increased persistence in the STEM workforce. Although much of the literature on undergraduate mentoring focuses on mentorship in research experiences, there is a growing recognition that additional mentoring relationships can support BBS students, and that the institutional context can influence academic mentoring. Therefore, it is crucial to understand: 1) the mentoring relationships that undergraduate BBS students are engaging in, 2) where and through what sources they receive mentorship, 3) the level of support they receive from their mentors, and 4) whether these trends differ by institution. To address these questions, we developed a mentoring-focused questionnaire, drawing from the Process-Oriented Model of Mentoring and the College Student Mentoring Scale, and distributed the questionnaire at two public, R1 institutions. We found that most BBS students at both institutions reported having a mentor. However, many of these students reported that their mentor was outside their institution or a peer mentor, emphasizing the role multiple mentors may have in the development of BBS students. Students reported a high level of perceived mentoring support in two areas: *Degree and Career Support* and *Existence of a Role Model*. Perceived mentoring support also correlated weakly with two aspects of a mentoring relationship: *interaction frequency* (weak negative correlation) and *relationship length* (weak positive correlation). This study provides a large, quantitative and descriptive analysis of BBS student mentoring relationships. Based on our results, covering a plethora of mentoring relationships, we provide recommendations directed to mentors, mentees, and mentoring program administrators. Our study also reveals a lack of evaluative tools that can be used for comparison of mentoring outcomes across institutions. Future research should focus on the development of mentoring-centric survey instruments that can be applied broadly across undergraduate BBS populations.

## Introduction

Mentoring plays an essential role in developing biology and biomedical science (BBS) students, because mentors provide students with opportunities for skill-building, help them reach degree milestones, and foster their affiliation to their disciplines and careers, leading to students’ increased persistence in STEM [1]. Existing literature suggests that mentored BBS students have higher grade point averages and more confidence in their career decision-making, than their non-mentored peers [2]. BBS students with mentors also show evidence of increased science identity and science efficacy [3]. Furthermore, BBS students with multiple mentors (faculty, postdoctoral researchers, etc.) demonstrate additive gains in their ability to think like a scientist and likelihood of enrolling in a PhD program [4]. Collectively, these studies highlight the value of mentoring relationships in promoting BBS students’ professional and personal development.

Mentoring structures for BBS students may emerge across a variety of relational and institutional contexts. Research mentors are among the most commonly studied mentoring relationships in the BBS literature [e.g., 3–5]. However, there is growing recognition of other mentoring relationships, such as peer mentors, who are undergraduate students that help provide support with course content, getting acquainted with the norms of their institution, and discussing personal challenges [6,7]. Undergraduate students may also receive mentoring support through career and professional development programs, which provide BBS students with specific advising of their degree progression and planning for next steps in their STEM careers [7]. Mentoring relationships may also extend beyond the academic institution, including family and friends, who provide BBS students with encouragement as they persist through their undergraduate careers. These mentoring relationships may differ in their structure, accessibility, duration, and the types of support they provide. Collectively, this highlights the need to better understand the broader network of mentoring relationships undergraduate BBS students engage in. To our knowledge, the presence of these different types of mentors has been primarily studied in the broader STEM context or in BBS graduate students [8,9], with limited exploration among undergraduate BBS students.

Despite their recorded benefits, mentoring relationships are unevenly distributed among BBS students. Many students lack mentorship because they do not know how to identify or approach a potential mentor [10]. Moreover, BBS students’ perceptions of mentor availability, perceived mentoring support, and mentor-mentee similarity also impact their ability to develop meaningful relationships with their mentors [11]. Beyond these individual factors, the institutional context, such as the departmental funding, faculty training and incentives, and curricular integration, likely plays a crucial role in determining both who gains access to mentorship and the quality of those relationships. One study found that faculty engagement in undergraduate research mentoring is strongly influenced by institutional supports like funding availability and reward structures [12]. To date, the measurable impacts of institutional differences on BBS students’ mentoring experiences remain underexplored. Therefore, investigating mentoring trends across institutions is a vital first step toward understanding how context shapes both access, quality, and characteristics of the mentoring relationship. These insights will be essential for designing targeted interventions that enhance mentoring support for BBS students.

Building on this gap, this study aims to investigate the types of mentors BBS students have access to and the characteristics of mentoring relationships at two different institutions. We employ an integrative approach that draws from two comprehensive models of mentoring to capture multidimensional mentorship experiences and potential institutional influences.

### Theoretical framework: Integrative approach

**Fig 1:**
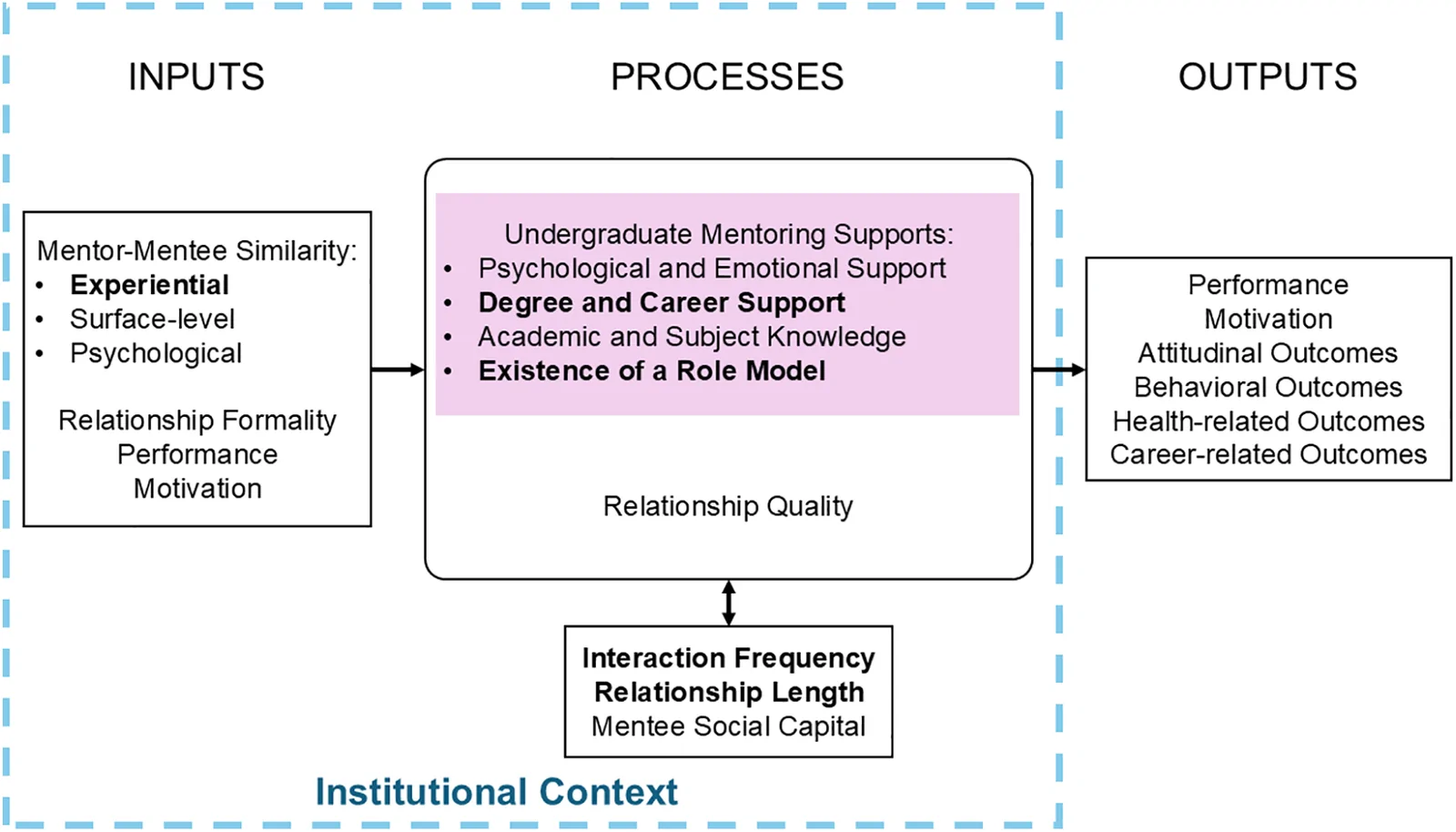
The process-oriented model of mentoring and its application in our study. Bolded items are variables measured in this study. The blue dashed line represents the institutional context surrounding a mentoring relationship and the shaded pink box represents the four supports in the Nora and Crisp model of undergraduate mentoring [18].

Research on undergraduate mentoring is guided by several theoretical frameworks, which include those adapted from the workplace literature [13,14] and models for student retention [15,16]. For the purpose of this study, we will be drawing from two complementary models, a process-oriented model [17] and a model for undergraduate mentoring support [18], to investigate the characteristics of mentoring relationships. Our integration of these models is summarized in Fig 1.

### Process-oriented model of mentoring

Eby and colleagues performed a meta-analysis of mentoring literature across an individual’s lifespan and concluded that there was a lack of consensus on the definition and operationalization of mentoring [17]. As a result, they developed a model that considers the relationship between several characteristics of a mentoring relationship. Drawing from McGrath’s original input-process-output model [19], Eby et al. conceptualize mentoring as three central processes: 1) *instrumental support*, the mentor behaviors that facilitate mentee goal achievement; 2) *psychosocial support*, the mentor behaviors that lead to the mentee’s personal and emotional development; and 3) *relationship quality*, the mentee’s perception of the mentor and evaluation of the mentoring relationship [17]. Influencing these processes are a variety of inputs and correlates of mentoring, which lead to measurable outputs.

The process-oriented model of mentoring, according to Eby et al. [17], situates four variables as potential inputs, or antecedents, that influence the processes of mentoring:

1. *Mentor-mentee similarity*, the degree to which a mentor and mentee are similar in three types of attributes. Similarity can be measured in *surface-level* attributes, such as race or ethnicity; *experiential* attributes, such as education, academic discipline, or affiliation; and *deep-level*, or psychological, attributes, which refer to the values, beliefs, and attitudes of the mentor and mentee. For BBS students, deep-level attributes, like personality, work and communication styles, have been documented to impact their mentoring experience [11].
2. *Relationship formality*, whether a mentoring relationship developed spontaneously (informal) or involved an organized matching process (formal). Formal mentors are well-documented in the literature, with BBS students showing a preference for structured mentor-mentee matching [10].
3. *Mentee performance*, a mentee’s competence in their area of work or their achievements. Among BBS students, this can be evaluated by their research-specific gains (e.g., technical research skills and techniques), professional gains (e.g., networking skills) and grade point average [2,17].
4. *Mentee motivation*, how much a mentee is involved in their work, which can be measured for BBS students as hours worked per week or level of involvement and persistence in a research project [2,17].

In addition to the antecedents of mentoring, this model discusses three correlates that can impact mentees’ perceived instrumental support, psychosocial support, and relationship quality:

1. *Interaction frequency*, which refers to the frequency of communication, number of contacts per month, or amount of time spent with the mentor. Frequent interpersonal communication is necessary for effective support and guidance. For example, undergraduate biomedical science students reported preferring monthly or more frequent mentor meetings [10]. Evidence suggests that more interaction frequency is positively correlated with mentees’ perceived instrumental and psychosocial support, although these correlations have not been studied among BBS students [17].
2. *Relationship length* refers to the amount of time since the mentoring relationship was established. The length of the relationship relates to both types of perceived mentoring support and perceived relationship quality, because they create more opportunities for learning and development. Previous work suggests that BBS students that engage in multiple semesters of mentored research experience higher research and professional gains that students who engage in only one semester [2].
3. *Mentee social capital*, the degree of support mentees obtain from their social network outside the mentoring relationship. Mentees with more mentors will have more social capital, which may increase the likelihood of receiving instrumental or psychosocial support. For example, BBS students that participate in a mentorship triad with a postdoctoral and faculty mentors are more likely to perceive they think and work like scientists than BBS students with only one mentor type [4].

Finally, Eby’s model defines the potential outputs, or consequences, of mentoring as: 1) *mentee performance*, 2) *mentee motivation*, 3) *attitudinal outcomes*, 4) *behavioral outcomes*, 5) *career-related outcomes*, or 6) *health-related outcomes*.

We consider the multifaceted nature of Eby’s model to characterize the relationship between BBS students and their mentors. To our knowledge, there is a lack of studies that explore several components of Eby’s model *simultaneously* in the context of BBS students. In this study, we directly measure experiential similarity, interaction frequency and relationship length, and instrumental and psychosocial support to characterize the relationship between BBS students and their mentors. To measure instrumental and psychosocial support, we draw from Nora and Crisp’s framework [18], which focuses on support provided to undergraduate students (Fig 1).

### Undergraduate mentoring supports

Nora and Crisp’s model [18] provides a framework for undergraduate mentoring support that integrates Kram’s mentoring theory [14] with established models of student success in higher education [15,16]. Grounded in an extensive review of the mentoring literature, their model emphasizes the multidimensional nature of mentoring and extends Kram’s original framework [14], by delineating instrumental and psychosocial support into four distinct domains. Here we describe each of these domains, and illustrate how mentors can provide forms of these supports to BBS students:

1. *Degree and Career Support* includes the assessment of the mentee’s skills and career planning. For instance, mentors of BBS students can provide this by sharing opportunities that allow them to demonstrate their skills, helping them build their professional networks, and encouraging them to explore options for graduate school [1,2,10].
2. *Academic and Subject Knowledge Support* refers to tutoring and the provisioning of strategies to enhance academic performance. For example, mentors may prepare BBS students to succeed in their STEM coursework by helping them identify their learning styles, reflect on their study habits and share study tips [6].
3. *Psychological and Emotional Support* involves mentors listening empathetically and offering encouragement. Mentors may provide this type of support to BBS students by showing empathy and concern for their feelings, making them feel welcome in the lab, and listening to their goals at the beginning of the research experience [1,2].
4. *Existence of a Role Model* captures how mentors exemplify behaviors that students can learn from. Mentors can be effective role models for BBS students, particularly when they share aspects of identity such as race or social class. This shared identity can enhance the mentoring relationship, fostering a sense of belonging and demonstrating that success in the field is attainable [20].

These constructs underpin the College Student Mentoring Scale (CSMS), an instrument designed to measure students’ perceptions of the mentoring support they receive [21]. The CSMS has been used in the past to evaluate mentoring support among nursing [22], first-generation [23], and first-year environmental science students [24]. The findings from these studies suggest that students perceive varying levels of support from their mentors, contributing to their success and persistence in college. However, many studies using the CSMS, including the ones referenced above, do not report validity evidence for the instrument. Efforts to present validity evidence on the constructs of the CSMS have been limited to a single community college [25] and a single Hispanic-Serving Institution (HSI) [21]. Collectively, these studies highlight the need to further explore the application of Nora and Crisp’s four-support model across disciplines, such as the biological and biomedical sciences, and to collect validity evidence at additional institutions [18].

### Conceptual synthesis

While Nora and Crisp’s model [18] identifies key domains of mentoring support within higher education, such as career guidance, psychosocial support, and role modeling, Eby et al.’s framework [17] emphasizes the relational dynamics, interpersonal quality, and contextual factors that shape how students perceive and experience support. Integrating these frameworks is important because mentoring cannot be fully understood by its functions alone; it also depends on the characteristics of the mentoring relationships, and institutional contexts in which it occurs. Together these models provide a more comprehensive view of mentoring, capturing both the *content of support* and the *relational and contextual conditions* that influence undergraduate student development, which is critical for assessing and designing effective mentoring programs.

### This study

Existing research has explored the experiences of mentored BBS undergraduates [2] but emphasizes mentoring through undergraduate research experiences [e.g., 2, 4, 26]. Thus, there is a need to explore the access that BBS students have to a broader range of mentoring relationships, including those with peers, professionals, and mentors outside of academic institutions (i.e., family and friends). Additionally, there is a need to explore across institutional contexts, 1) how BBS students access these various mentoring relationships across different contexts, 2) the characteristics of these mentoring relationships, and 3) the relationship between these characteristics and BBS students’ perceived mentoring support. In this study, we were interested in the mentoring relationships that undergraduate BBS students may be engaging in at two different public institutions. We integrate two frameworks that, to our knowledge, have not been previously applied to the BBS student context *simultaneously* and provide quantitative insight into these students’ mentoring relationships, answering the following research questions:

1. What proportion of undergraduate BBS students have a mentor at Institution A and Institution B?
2. Where (inside or outside of the institution) and through what sources (peer mentors, organized mentoring programs) do students report receiving mentorship?
3. How do undergraduate BBS students perceive their mentoring relationships, in terms of mentor-mentee experiential similarity, interaction frequency, relationship length, and degree of perceived mentoring support?
4. How are mentor-mentee interaction frequency and mentoring relationship length related to undergraduate BBS students’ perceived level of mentoring support?

## Methods

### Participants and data collection

Data presented in this study is part of a larger dataset collected in the Spring 2024 semester. Approval from the Florida International University Institutional Review Board was acquired before beginning this study. Written consent from all participants was collected as an electronic form. All data were collected at two institutions: 1) a large, Predominantly Hispanic R1 institution in the southeastern United States (Institution A); and 2) a large, Predominantly White R1 institution in northeastern United States (Institution B). Table 1 provides an overview of different aspects of these institutions. First, 18 cognitive interviews [27] were performed at the start of the Spring semester of 2023 to evaluate the wording of the questionnaire items, collecting evidence of response process validity [28]. Based on the results from the cognitive interviews, the questionnaire was refined, and a pilot questionnaire was administered later in the same semester. In the Spring 2024 semester, the questionnaire was revised based on pilot results and administered at both institutions. The questionnaire was administered to students enrolled in core biology courses (e.g., General Biology I and II, Cell Biology, Genetics, and Evolution) and electives with students from various college academic levels (freshman, sophomore, junior, and senior) that contained both biology majors and non-biology majors. We use the term Biology and Biomedical Sciences (BBS) students to refer to all students who are enrolled in the biology courses used for recruitment due to their varying majors. The questionnaire was distributed through Qualtrics (online survey software; Provo, UT and Seattle, WA) via email from course instructors and in-class visits during the last third of the semester, from March 2024 to May 2024. Instructors were given the opportunity to opt into compensating students for participation through extra credit. In total, 1,327 students at Institution A completed the questionnaire, and 1,174 students at Institution B completed the questionnaire. The demographics of participants are shown in Table 2.

**Table 1:** Overview for each institution in our study.

Institutional Aspect	Institution A	Institution B
Type of Institution	Public, Research (R1), Predominantly Hispanic	Public, Research (R1), Predominantly White
Total Undergraduate Enrollment	44,045	20,463
Students Who Commute to Campus	92%	67%
Student-Faculty Ratio	21:1	11:1
Pell Grant Eligibility	46%	34%
First-Generation Status	33%	18%

**Table 2:** Student demographics for both institutions.

Institution A, n = 1,327			Institution B, n = 1,174		
**Race/Ethnicity**	**n**	**%**	**Race/Ethnicity**	**n**	**%**
Hispanic	974	73.4	Hispanic or Latino	111	9.5
White	86	6.5	White	541	46.1
African American	114	8.6	Black or African American	107	9.1
Asian or Pacific Islander	74	5.6	Asian	260	22.1
American Indian/Alaskan Native	2	0.2	Native Hawaiian	1	0.1
Non-Resident Alien	56	4.2	American Indian/Alaska Native	4	0.3
Not Reported	8	0.6	International (Non-Resident Alien)	47	4
No Data	13	1	Two or more races	48	4.1
			Unknown	42	3.6
			No Data	13	1.1
**Year in School**	**n**	**%**	**Year in School**	**n**	**%**
College Freshman (0–29 units)	153	11.5	College Freshman (0–29 units)	272	23.2
College Sophomore (30–59 units)	274	20.6	College Sophomore (30–59 units)	350	29.8
College Junior (60–89 units)	419	31.6	College Junior (60–89 units)	199	17
College Senior (90 + units)	468	35.3	College Senior (90 + units)	292	24.9
No Data	13	1	Other	48	4.1
			No Data	13	1.1
**Career Goal**	**n**	**%**	**Career Goal**	**n**	**%**
Health Sciences	950	71.6	Health Sciences	848	62.6
Other STEM	105	7.9	Research	153	11.3
Animal Science	86	6.5	Other STEM	76	5.6
Research	79	6	Animal Science	31	2.3
Non-STEM	69	5.2	Non-STEM	12	0.9
Unsure	38	2.9	Unsure	54	4
**Major**	**n**	**%**	**Major**	**n**	**%**
Biological Sciences	786	59.2	Biological Sciences	250	21.3
Psychology	113	8.5	Biomedical Science	208	17.7
Computer Science	38	2.9	Neuroscience	119	10.1
Natural & App. Sciences	36	2.7	Traditional Nursing	93	7.9
Biochemistry	30	2.3	Pharmacy	71	6
Behav. Neuroscience	27	2	Psychology	40	3.4
Marine Biology	24	1.8	Exploratory Bachelor	22	1.9
Crime Science	18	1.4	Environmental Engineering	20	1.7
Biomedical Engineering	16	1.2	Public Health	19	1.6
Interdisciplinary Studies	16	1.2	Exercise Science	17	1.4
Liberal Studies	15	1.1	Nutrition Science	17	1.4
≤ 12 students/ major	208	15.7	Pharmaceutical Sciences	17	1.4
No data	13	1	Post-Bacc. Program – Medical School Non-Degree	15	1.3
			Undergraduate Non-Degree	14	1.2
			Industrial Engineering	12	1
			≤ 11 students/major	227	19.3
			No data	13	1.1

### Mentoring questionnaire

*Preliminary mentoring questions.* To determine which students had access to mentors, they were provided with a series of screening questions. First, participants were given the following definition of a mentor: “A mentor is a person who supports, advises, and guides you.” We purposefully chose a broad definition of a mentor to capture a variety of mentoring relationships from which our students may obtain support. Although concise, this definition reflects core functions of mentoring described in prior literature, including the provision of guidance and developmental support [14,18]. Then, they were asked to indicate whether they had a mentor in any of four categories: 1) at the institution, 2) outside the institution, 3) peer mentors, or 4) an organized mentoring program. Participants were able to select multiple categories, as these were not mutually exclusive and were intended to capture different dimensions of mentoring relationships (e.g., context, role, and structure). These categories were intended to capture broad mentoring contexts and structures rather than directly operationalize mentoring formality as a multidimensional construct. Accordingly, percentages reported for each category reflect the proportion of students who identified at least one mentor in that category and do not sum to 100%. Additionally, students had the option to select if they had more than one mentor in their lives (Table S1 in S1 File).

*Mentoring antecedents and correlates.* We measured antecedents and correlates of mentoring, such as: experiential similarity, interaction frequency, and relationship length, as described in Eby et al [17]. To start, we asked students to choose a mentor that has most influenced their career development and to keep this mentor in mind as they answered questions related to experiential similarity, interaction frequency, and relationship lengths.

To measure experiential similarity, we asked students to identify if they shared their intended career field with their chosen mentor (Table S1 in S1 File). Interaction frequency was measured by a single multiple-choice question that asked students to select how often they met with their mentors by selecting one of six options, ranging from “Daily” to “Less than once a month” (Table S1 in S1 File). Relationship length was also measured with a single multiple-choice question that asked students to indicate how long they were working with their mentor by selecting one of seven options, ranging from “Less than one month” to “More than four years” (Table S1 in S1 File).

*Mentoring support.* We used adapted items from the CSMS to measure students’ perceptions of the mentoring support they received from their mentors. The CSMS measures four types of support relevant to undergraduate mentoring: 1) *Psychological and Emotional Support*; 2) *Degree and Career Support*; 3) *Academic and Subject Knowledge Support*; and 4) *Existence of a Role Model* [18,21]. However, we chose to only include items related to *Degree and Career Support (DCS)* and *Existence of the Role Model (ERM)* because students expressed during the cognitive interviews (Spring 2023) that these were the most relevant to their mentoring experiences. These two supports also align with our overarching goal of learning about students’ career development, and we were confident that the two-factor assessment could provide valuable information about our student population (Table S2 in S1 File). We used four DCS items and six ERM items, and a six-point Likert scale (1 = Strongly disagree to 6 = Strongly agree) was used to rate these items.

### Data analysis

Descriptive statistics. Following questionnaire distribution, we ran descriptive statistics to get a sense of the different measures of mentoring antecedents and correlates (experiential similarity, interaction frequency, and relationship length) and mentoring supports (*Degree and Career Support* and *Existence of a Role Model*) across institutions. For questions in the Preliminary Mentoring and Mentoring Antecedents and Correlates sections, Fisher’s Exact test was used to determine potential significant differences between institutions by individual category. Comparative analyses of these measures were run using Python *v3.11.11* and R version *v4.3.2* [29].

Instrument validity and reliability. To determine the construct validity of the mentoring support scales of the questionnaire, we conducted: 1) factor analyses to determine the factor structure of the CSMS subscales and 2) tests of measurement invariance to determine if the factor structure of the CSMS could be applied to both institutions. These tests of validity were applied specifically to the CSMS because of its multidimensional nature, and not to the unidimensional constructs of experiential similarity, interaction frequency, and relationship length. Prior to conducting the factor analyses, we tested for key assumptions including multivariate and univariate normality, collinearity, and examined the impact of outliers and patterns of missing data. Assumption testing for confirmatory factor analysis (CFA) ensured that the model’s estimates are accurate and interpretable.

Since we drew items from an existing questionnaire (CSMS), we applied independent CFAs for Institution A and B. We specified a two-factor CFA, with four items corresponding to the *DCS* scale and six items representing the *ERM* scale (Table S2). Multiple fit indices (chi-square value from the robust MLR [MLR χ^2^]; comparative fit indices [CFI]; the root-mean-square error of approximation [RMSEA]; and the standardized root-mean-square residual [SRMR]) were all used to evaluate the model fit. Using Hu and Bentler’s recommendations [30], we used the following criteria: CFI > 0.95, SRMR < 0.08, and RMSEA < 0.06. The reliability of the mentoring items was also evaluated by examining McDonald’s omega coefficient (**ω**_**t**_) for each subscale with values > 0.70 considered acceptable [31]. R packages *psych v.2.4.12* [32] and *lavaan v.0.6.19* [33] were used for these analyses.

Next, tests of measurement invariance were conducted to evaluate whether the two-factor model could be meaningfully compared across both institutions (i.e., *Do the CSMS mentoring constructs carry the same interpretation for students at Institution A and B?*). We conducted a series of three sequential tests, each assessing a different assumption of invariance [34,35]. The first step, configural invariance, assessed whether Institution A and B students broadly conceptualize the support from their mentors in a similar manner. An assumption of this test is that the same factor structure holds for both institutions, while allowing the factor loadings and item intercepts to vary across groups. To achieve this test, we assessed the factor structure for each group using the same goodness of fit indices used for the CFAs (MLR χ^2^, CFI, SRMR, RMSEA) [36]. The second step, metric invariance, assessed whether BBS students at Institution A and B were interpreting individual questionnaire items in the same way. This step tested the assumption that questionnaire items contributed to each construct in a similar manner across groups. We identified a metric model that constrained the factor loadings to be equal across groups, while allowing the item intercepts to vary [37,38]. To achieve metric invariance, this metric model was compared to the configural model by assessing changes in chi-square (∆χ^2^) and changes in model fit indices (∆RMSEA, ∆SRMR, and ∆CFI) [39]. If the cutoffs for these changes were within the appropriate range, (∆RMSEA <0.015, ∆SRMR <0.030, ∆CFI < 0.010), then metric invariance was achieved [40]. Due to the sensitivity of chi-square to large sample sizes, we chose to rely on model fit indices to test this step [41,42].

A test for scalar invariance was conducted to evaluate if BBS students at Institution A and B were using the response scales for the items similarly. This test eliminates the influence of systematic biases on observed differences in students’ responses and is essential for comparing latent means across groups [43]. To assess scalar invariance, item intercepts were constrained to be equal across groups, and the fit statistics of the scalar model were compared to those of the metric model using changes in chi-square (∆χ^2^) and model fit indices (∆RMSEA, ∆SRMR, ∆CFI), with standard cutoffs of ∆RMSEA < 0.015, ∆SRMR < 0.010, and ∆CFI < 0.010). Achieving all three tests of measurement invariance would ensure that the latent means of the mentoring supports could be compared across institutions. Measurement invariance tests were run using R package *lavaan v0.6.19* [33].

*Correlational analysis*. In order to determine the relationship between the mentoring supports (*DCS* and *ERM*) and interaction frequency or relationship length, we conducted individual tests for Spearman’s correlation. All correlational analyses were run in Python *v3.11.11*, using *scipy v1.13.1* [44].

## Results

### Proportion of mentorship, source of a mentor, and mentoring relationships at each institution

**Fig 2:**
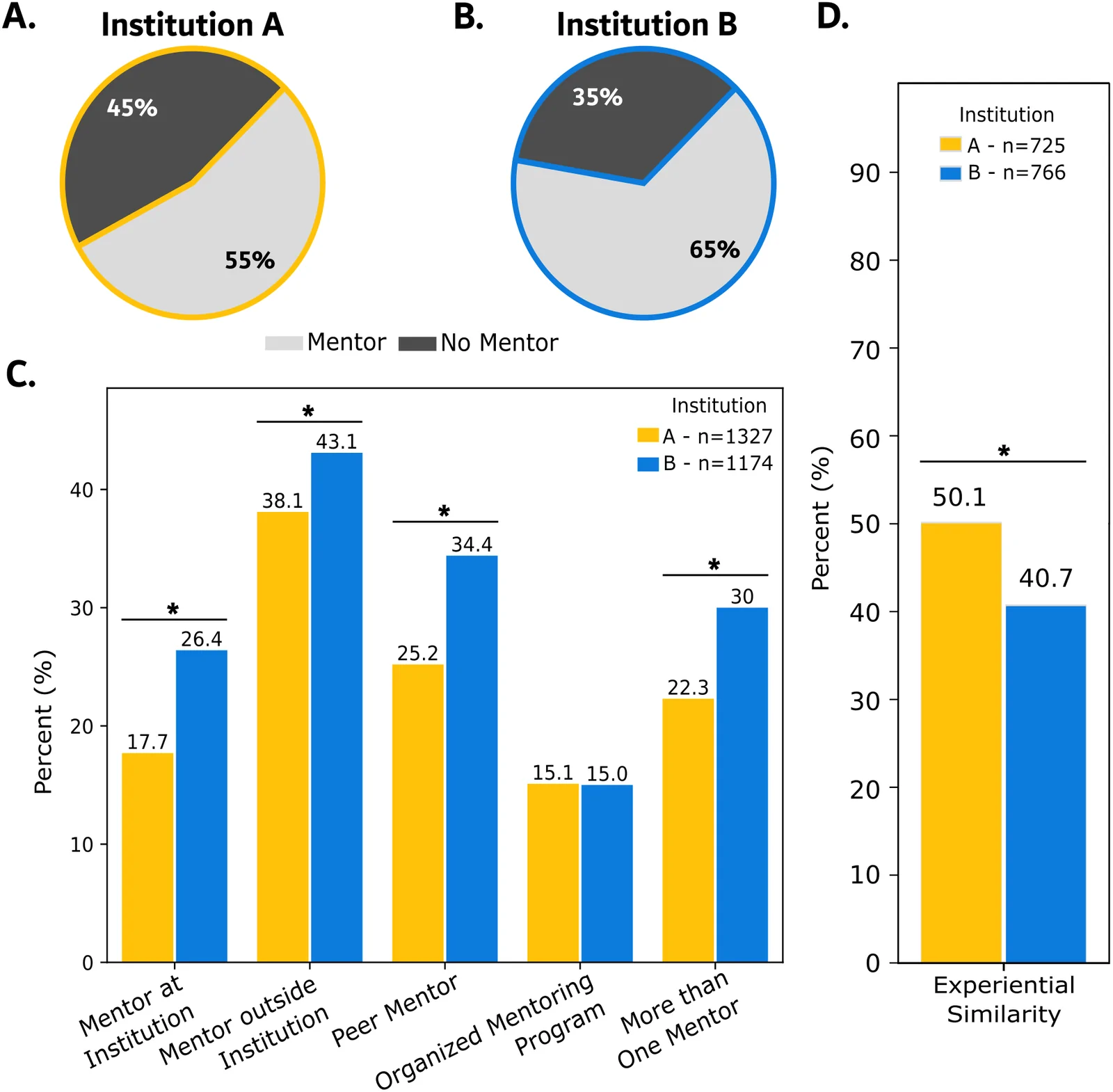
Mentors of undergraduate BBS students. **(A)** Proportion of students who reported having a mentor at Institution A (n = 1,327), **(B)** Proportion of students who reported having a mentor at Institution B (n = 1,174), **(C)** Proportion of students that reported each source of mentorship by institution, and **(D)** Proportion of students that reported sharing their intended career field with their mentor. Asterisks indicate significant differences between institutions (p < 0.05, Fisher’s Exact).

More than 50% of students at both institutions reported having a mentor. However, a significantly lower proportion (55%) of students reported having a mentor at Institution A (Fig 2A) than at Institution B (65%) (Fig 2B) (p = 6.92 × 10^−8^). BBS students at both institutions most commonly reported mentors “Outside of the institution” (38.1% Institution A, 43.1% Institution B) (Fig 2C). We also observed significant differences in the proportion of students reporting each source of mentorship across institutions. Specifically, students at Institution B reported significantly higher proportions of students reporting peer mentors as a source of mentorship (25.2% Institution A, 34.4% Institution B) (p = 3.36 × 10^−7^) and mentors within the institution (17.7% Institution A, 26.4% Institution B) (p = 1.12 × 10^−7^) than students at Institution A (Fig 2C). Reported experiential similarity (career field) was high at both institutions (Fig 2D), although a significantly higher proportion of students at Institution A (50.1%) reported this type of similarity than at Institution B (40.7%) (p = 3.27 × 10^−4^).

**Fig 3:**
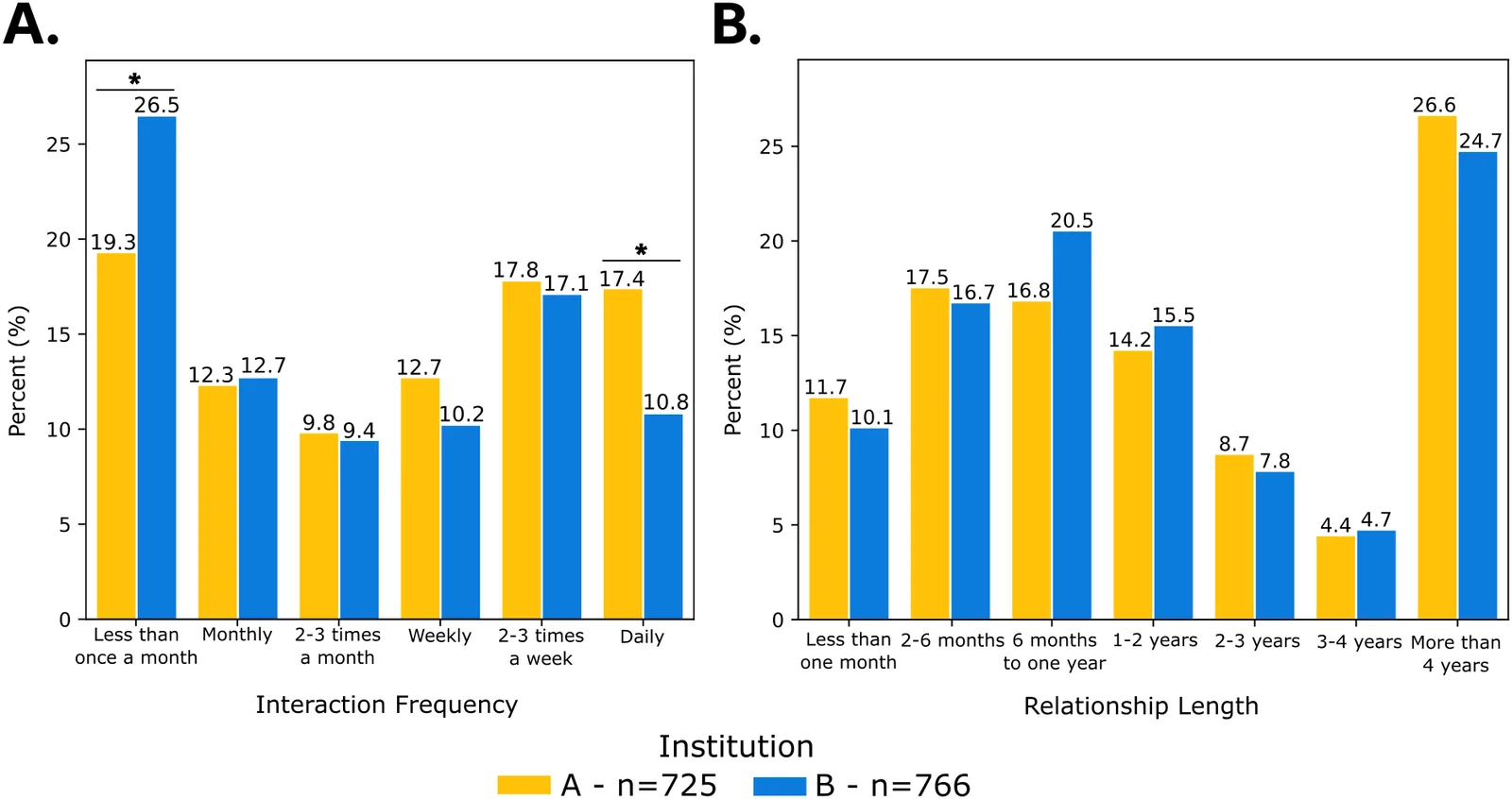
Proportion of students by the correlates of mentoring. **(A)** Frequency of interaction with their mentors, and **(B)** Length of relationship with their mentors. Asterisks indicate significant differences between institutions (p < 0.05, Fisher’s Exact).

The interaction frequency that was most reported for both institutions was “Less than once a month,” although this option was significantly higher (p = 0.001) in the Institution B sample (19.3% Institution A, 26.5% Institution B) (Fig 3A). Institution A students also reported meeting with their mentors “Daily” significantly more than Institution B students (17.4% Institution A, 10.8% Institution B) (p = 3.26 × 10^−4^). The relationship length that was most chosen in both institutions was “More than four years” (26.6% Institution A, 24.7% Institution B) (Fig 3B). However, there were no significant differences between institutions for relationship length.

### A two-factor model accounts for perceived mentoring support in the *Degree and Career Support* and *Existence of a Role Model* domains

Items from the CSMS had means that ranged from 4.98 to 5.36 for Institution A and means between 4.75 to 5.2 at Institution B. Standard deviations ranged from 0.96 to 1.23 across both institutions (Table S2 in S1 File). Assumption testing conducted prior to the CFAs demonstrated high negative skewness across institutions, with values ranging from −2.24 to −1.08. Kurtosis ranged from 3.66 to 7.07. Our sample demonstrated a lack of multivariate normality, per the results from Mardia’s test of multivariate normality (p < 0.001). Mahalanobis distance identified 875 multivariate outliers (p < 0.01) across the full sample. Visual inspection of boxplots suggested that extreme values reflected genuine differences in students’ mentoring experiences rather than data entry or measurement errors. In addition, Wilcoxon rank-sum tests indicated no significant differences between outlier and non-outlier cases (p > 0.05). Given these findings, all cases were retained to preserve the full variability of students’ responses for subsequent analyses. Across both institutions, less than 1% of the data was missing. These collective findings suggest non-normality of the data, and therefore we used a robust maximum likelihood ratio (mlr) as an estimator for the CFAs and weighted least squares mean and variance adjusted (WLSMV) for the measurement invariance tests.

The fit of the *Degree and Career Support* (DCS) and *Existence of a Role Model* (ERM) subscales of the CSMS was evaluated using individual CFAs for Institution A and B. Initial models demonstrated suboptimal fit, particularly for Institution A, whereas Institution B showed a comparatively better fit. (Table S3 in S1 File). Inspection of the modification indices suggested that dropping two items (ERM1, ERM5) would improve model fit for both institutions. We felt confident removing these items based on conceptual and qualitative considerations. Item ERM1 (“Shares personal examples of difficulties he or she had to overcome to accomplish academic goals”) was removed based on cognitive interview data indicating that students found the item irrelevant to their mentoring relationships. Item ERM5 (“I look up information regarding college-related issues”) was removed due to its conceptual overlap with *Degree and Career Support* items. We considered that the reduced scales still captured the conceptual breadth of the constructs and determined that the remaining items continued to represent the key dimensions of *Degree and Career Support* and *Existence of a Role Model*. Additionally, a correlated error term was added between items DCS2 and DCS4, which both measure how a mentor supports students in examining their career options. After these adjustments, model fit improved for both Institution A and Institution B (Table S3 in S1 File).

The final two-factor solution consisted of items DCS1, DCS2, DCS3, and DCS4 representing *Degree and Career Support*, and items ERM2, ERM3, ERM4, and ERM6 representing *Existence of a Role Model*. Standardized factor loadings were generally above 0.70, indicating that for most items, more than 50% of the variance was explained by the latent factors. McDonald’s omega reliability coefficients were acceptable across institutions (Table S4 in S1 File). The correlation between the *DCS* and *ERM* factors was strong: 0.86 for Institution A and 0.80 for Institution B (Fig S1 in S1 File). Although the factors were strongly correlated, discriminant validity was supported by a heterotrait-monotrait ratio (HTMT) value of 0.86, which falls below the 0.90 threshold recommended for applied social science research [45].

With validity evidence for the two-factor model collected, we were able to analyze the distribution of responses for each CSMS subscale. Overall, BBS students at both institutions reported high levels of mentoring support in the *Degree and Career Support* factor with a mean of 5.11 for the Institution A and 4.9 for the Institution B (Fig S2A in S1 File) and in the *Existence of a Role Model* factor with a mean of 5.19 for Institution A and 5.08 for Institution B (Fig S2B in S1 File).

### Measurement invariance: Testing the two-factor model across institutions

Due to the reasonable fit of the two-factor model for both institutions, we proceeded with measurement invariance testing using a series of nested models. Beginning with the configural model as a baseline, we compared the Institution A and B groups by progressively adding constraints. We found support for configural and metric invariance, but not for scalar invariance. The scalar model exhibited a decline in model fit (e.g., ∆RMSEA = +0.023), exceeding recommended thresholds for change in fit indices. Fit statistics for each level of invariance are summarized in Table S5 in S1 File. These findings suggest that the constructs were conceptualized similarly by students at both institutions and that the items functioned equivalently in terms of factor loadings. Since scalar invariance was not supported, mean differences across institutions should be interpreted cautiously, as they may reflect group response patterns rather than true differences in the mentoring constructs. However, correlations among the constructs can still be meaningfully interpreted, because correlations reflect the pattern of associations rather than absolute mean levels, offering insight into how mentoring supports relate to other outcomes [46].

### Correlation between mentoring supports and interaction frequency or relationship length

**Fig 4:**
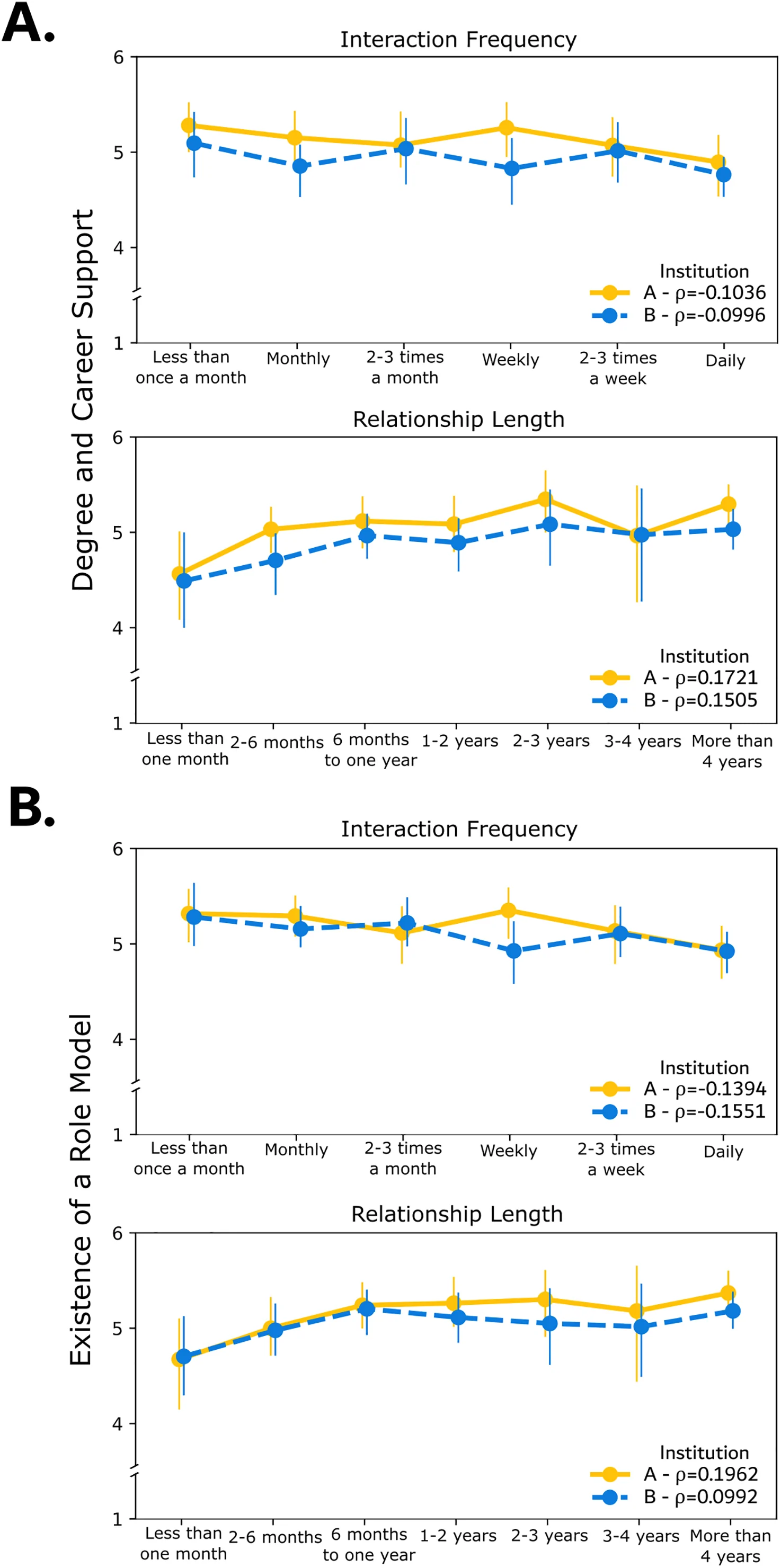
Mentoring supports by mentor-mentee interaction frequency and relationship length. **(A)** *Degree and Career Support*, and **(B)** *Existence of a Role Model*. Institution A results are indicated by a yellow, solid line, while Institution B results are indicated by a blue, dashed line. Spearman’s rho [ρ] values are indicated for each correlation.

With metric invariance established, correlational analysis between the means of the mentoring support scales and additional measures of mentoring (interaction frequency and relationship length) was appropriate. Both *DCS* and *ERM* had a weak negative correlation with interaction frequency (Fig 4 A and B). Overall, students who meet with their mentor “Less than once a month” perceived they were receiving the most mentoring support, although students largely reported high scores of both types of support, regardless of the interaction frequency. Relationship length had a weak positive relationship with both types of support measured (Fig 4 A and B). However, the level of *Degree and Career Support* obtained drops (for Institution A students) or stagnates (for Institution B students) at the “3–4” year mark of the mentoring relationship. On the other hand, support in the *Existence of a Role Model* domain stagnates near the “1–2 year” mark, especially for Institution B students. Due to the range of mentoring relationships captured in this study, we also performed a correlational analysis with only the subset of students that reported mentors at their institution (Fig S3 in S1 File). We observed similar results, where interaction frequency had a weak negative correlation to both mentoring support scales, and relationship length a weak positive correlation to both supports.

## Discussion

In this study, we aimed to better understand the access and characteristics of mentoring relationships of biology and biomedical science (BBS) students at two institutions, representing two different student demographic groups, through a quantitative lens. We collected cross-sectional data from the perspective of all BBS undergraduates, not just those participating in undergraduate research experiences or targeted mentoring programs. With Nora and Crisp’s undergraduate mentoring theory [18] and the process-oriented model developed by Eby and colleagues [17] being two of the dominant frameworks around which undergraduate mentoring studies are designed, we were particularly interested in how these characteristics and supports varied across institutional contexts. In what follows, we contextualize key findings.

### Most BBS students have a mentor

Our first research question was focused on the proportion of BBS students that have access to mentorship. It is encouraging that most students at both institutions report having a mentor (Fig 2 A and B). Strada-Gallup reported that 43% of over 5,000 college graduates across the U.S. reported having a mentor during their undergraduate studies [46]. The proportion of students that reported having a mentor at both institutions studied was higher than the nationwide results. However, given the importance of mentoring in academic environments [1–4], we would want the proportion of mentored BBS students to be much closer to 100%.

In our data, the proportion of students reporting a mentor at Institution A was significantly lower than that at Institution B (Fig 2 A and B). Although comparative studies of mentorship practices at predominantly Hispanic (such as HSIs) and predominantly White institutions (PWIs) have not been studied extensively, a case study of an HSI found that only 23% of students reported having a mentor [47], which emphasizes the relatively low mentorship participation rates that exist at HSIs. While our data shows over half (55%) of Institution A students reporting a mentor (Fig 2A), it does support the narrative within the literature of Hispanic students engaging in mentoring at lower rates [48]. There are several factors that contribute to this difference. First, institutional structures and resources such as student‑to‑faculty ratios, availability of mentoring programs, and advising support often differ between predominantly Hispanic and predominantly White institutions. HSIs tend to receive less funding and operate with more constrained resources for student support services compared to other institutions, which can limit the scope and consistency of mentoring and advising available to students [49,50]. Second, demographic and cultural dynamics, including the availability of mentors who share students’ ethnic, gender, or disciplinary identities, can influence perceptions of support and role modeling [51]. These findings suggest that the lower mentoring reported at Institution A may reflect differences in institutional structures and mentoring relationships, highlighting the role of context in shaping students’ mentoring experiences.

### Most BBS undergraduates report having a mentor outside of their academic institution

Whether BBS students report having a mentor could be tied to their career interests. Our data show that over 70% of undergraduate BBS students at both institutions reported a career goal in the health sciences (Table 2), which may encourage these students to search for non-academic or clinical mentors outside of their academic environment. Consistent with this idea, we observed that the most commonly reported source of mentorship is “outside the institution” (Fig 2C). Prior research suggests that mentoring relationships outside academic institutions may emerge through a variety of personal, professional, and community-based contexts [52]. For example, one study of postdoctoral researchers has emphasized the importance of having multiple mentors, including mentors outside of academia, in preparation for entering the non-academic workforce [53]. Our findings suggest that undergraduate BBS students may seek mentoring support through multiple pathways beyond their academic institution. Instead, the role of faculty may be to serve as “guideposts” that encourage and lead undergraduate BBS students to mentors that can provide the needed non-academic support. This model of faculty engagement with mentoring would lessen the mentoring burden on individual faculty, while ultimately contributing valuable support for undergraduate students with career goals beyond academia. Concordantly, students in our study also report having more than one mentor (Fig 2C).

### BBS undergraduates report engaging in peer mentoring

We observed that more than 25% of BBS students report having peer mentors (i.e., students who mentor other students), especially at Institution B (34%) (Fig 2C). While we collected no additional data related to peer mentoring, these results suggest that undergraduate BBS students report peer mentors as a source of mentorship more frequently than mentors at their institution. This is not surprising given the variety of methods through which undergraduate STEM students have access to peer mentoring. Students can engage with peer mentors through a variety of institutional or interpersonal contexts, including undergraduate research experiences [54], disciplinary programs [55], courses [56,57], or through spontaneous interactions. Our results make sense given the presence of peer mentoring programs at both institutions, including Learning Assistants and Peer-Led Team Learning at Institution A and Peer Assisted Learning at Institution B.

Collectively, our data suggest that undergraduate BBS students seek mentoring from multiple people, not just faculty advisors or instructors, and could serve as a foundation for reconceptualizing how we provide mentoring to BBS undergraduates from an institutional level. The mentoring commitment for faculty, particularly those at research-intensive institutions, could be decreased by focusing on 1) helping students identify mentors outside the institution and 2) increasing support and visibility of peer mentoring programs.

### Nearly half of all BBS undergraduate respondents reported sharing their career field with their mentor

We observed that nearly half of the students in both institutions reported sharing their career field with their mentor (Fig 2D). This suggests that the other half of surveyed BBS students consider someone not in their career field as the mentor most influential to their career development. Previous studies suggest that psychological similarity, not measured in our study, and experiential similarity, represented in our data by a shared career field, might be more influential on mentoring outcomes than surface-level similarity [17,58], which emphasizes the need to increase undergraduate student access to mentors in their intended career field. Interestingly, Institution A students reported greater experiential similarity with their mentors than students at Institution B (Fig 2D). In our sample, Institution A students are less “Unsure” about their career goals, which could lead to more targeted searches for a mentor (Table 2).

### We collected validity evidence for the *Degree and Career Support* and *Existence of a Role Model* subscales of the CSMS at both institutions

We conducted separate confirmatory factor analyses (CFAs) for both the Institution A and B samples, each demonstrating acceptable model fit for the CSMS subscales. These results indicate that the factor structure of the CSMS is consistent within each institutional context, suggesting that the assessments measure the intended constructs. However, subsequent multi-group analyses revealed that full measurement invariance was not achieved. Although we found support for configural and metric invariance of the two CSMS subscales, we did not achieve scalar invariance. Thus, we could not compare the means of mentoring supports but could confidently conduct correlational analyses between these groups. One possible explanation for the lack of scalar invariance is differential item functioning influenced by institutional culture and emphasis on mentorship. For instance, students at Institution A may interpret and respond to items differently than those at Institution B due to variations in institutional support and emphasis around mentorship. Our results align with findings from Crisp, who reported significant differences in the CSMS factor structure between White and Hispanic students at an HSI, indicating potential measurement non-invariance across ethnic groups [25]. Future research should consider employing cognitive interviews to explore how students from different institutional backgrounds interpret CSMS items.

### Interaction frequency and relationship length did not correlate strongly with BBS students’ perceived mentoring support

Our work also explores how additional aspects of mentoring relationships, such as interaction frequency and relationship length, are correlated with students’ perceived mentoring support.

*Interaction frequency*. Our data suggests that a number of undergraduate BBS students are interacting with their mentors very frequently (at Institution A) or very infrequently (at Institution B) (Fig 3A). One possible explanation is that the type of mentoring relationship may influence interaction frequency. Previous research suggests that students may engage with mentors outside academic settings, such as relatives or family friends [52], which could be associated with more frequent interactions. Institution A has a higher proportion of students who commute to campus than Institution B (Table 1), which may lead to more frequent interactions with their mentors outside their institution. However, because we did not directly distinguish between specific characteristics of mentoring relationships (e.g., formality or role), this interpretation should be considered speculative. Other institutional contextual variables, such as the student-faculty ratio and proportion of first-generation students may influence interaction frequency as well and should be the subject of further study.

Correlational analysis revealed weak negative interactions between interaction frequency, and both *DCS* and *ERM* across institutions (Fig 4 A and B). We obtained similar results when repeating these analyses for students who reported their mentor was at their institution (Fig S3 in S1 File). This is surprising given previous findings suggesting that more frequent mentor-mentee interactions are generally associated with higher mentee perceptions of instrumental (i.e., career) and psychosocial support [17]. One potential explanation is that the quality or focus of interactions may influence mentoring support more than frequency alone. Students who meet with their mentors less than once a month may have more intentional, goal-focused meetings, whereas students who meet weekly may engage in routine check-ins that do not always translate into higher perceived support. Mentor-mentee similarity in characteristics such as gender, ethnicity, or disciplinary background may further moderate these effects. For example, a study of Hispanic STEM students found that interaction frequency strengthened mentee satisfaction with support only when mentors shared similar demographic characteristics [59]. A second hypothesis is that students who interact frequently with their mentors may be having more conversations about their professional goal setting (*DCS*), than asking their mentor for advice on how to overcome obstacles (*ERM*). Previous literature suggests that students who spend more time with their peer mentors reported higher career-related support, but not role modeling support [60,61]. Together, these results suggest that less frequent, targeted mentoring interactions may sometimes be perceived as more valuable, highlighting the importance of examining not only the frequency, but also the content of mentoring meetings. Future research should investigate how interaction frequency, mentor characteristics, and focus jointly influence perceived mentoring support across multiple dimensions.

*Relationship length*. Around a quarter of the students surveyed at both institutions reported mentoring relationships that were longer than four years (Fig 3B), which may reflect long-standing mentoring relationships that began prior to college (i.e., family, community members). However, because specific characteristics of mentoring relationships (e.g., formality or role) were not directly measured, this interpretation should be made with caution. The correlation between relationship length and students’ perceived *DCS* and *ERM* was also weak across institutions. This suggests that even students with more than four years of mentorship experience did not report receiving more mentoring support than students with less than one month of mentorship. This seems counterintuitive, as one would expect that long-term relationships would create more opportunities for rapport building and the provisioning of mentoring support. One explanation for this pattern is that long-term mentoring relationships may not always indicate that high-quality mentoring is taking place. It is possible that mentored BBS students may not be satisfied with their mentoring relationships and thus do not report receiving higher levels of mentoring support. Eby et al. found that relationship quality, instrumental, and psychosocial support positively correlated with each other [17]. However, given that we observed high perceived mentoring support regardless of relationship length, the interaction between relationship quality, mentoring support, and relationship length may be more complex. Future work may want to measure BBS students’ satisfaction of mentoring relationships and explore its connection to relationship length.

### Implications for practice

To our knowledge, this is the first study to make direct comparisons of mentoring experiences for undergraduate BBS students across institutional contexts. The results of this cross-sectional study describe student mentoring relationships and discuss correlations between specific aspects of the mentoring relationship and degree of mentoring support obtained. These data inform recommendations for mentoring practices that cater to the needs of BBS students at two different institutions. Based on our findings, we provide recommendations to encourage the development and strengthening of mentoring relationships in these contexts (Table 3).

**Table 3:** Recommendations for mentorship practice across stakeholders.

Stakeholder	Recommendations
Mentoring Program Administrators	Encourage students to seek mentorship within the institution, in addition to mentors outside. Communicate the benefits of having a mentor for BBS students’ personal and professional development.
Mentors	Serve as “guideposts” that direct mentees to resources and other mentors for more directed support. Focus on the quality of mentorship (i.e., types of mentoring support provided) over the quantity of meetings with students. Strengthen mentoring relationships by sharing aspects of your identity that may resonate with mentees.
Mentees (i.e., students)	Capitalize on mentoring programs that exist in your institution that can provide you with tailored support for your major, college or career. Seek a diverse network of mentors (e.g., faculty, family, peers) that can provide support during your undergraduate career.

### Limitations and future work

Although our study provides a large quantitative analysis of undergraduate BBS student mentoring relationships across institutional contexts, there are some limitations to our work. First, all measures are based on student self-report, which may introduce bias in perceptions of mentoring support and relationships. In addition, the cross-sectional design of this study limits our ability to draw causal inferences about how mentoring relationships develop or influence student outcomes over time. We are also unable to obtain data that would further describe the institutional contexts in which the mentoring relationships occur such as class size, advising structures, or programmatic supports, limiting our interpretations of observed institutional differences. These differences may be influenced by contextual factors not measured in this study, and future research should examine how such variables shape mentoring experiences.

In this study, we asked students to report whether they had mentors at their institution or outside it, providing valuable data showing that students seek out non-academic mentors at both Institution A and B. However, our measures captured broad sources of mentorship (e.g., within the institution, outside the institution, peer mentors, organized programs) rather than distinguishing specific characteristics of mentoring relationships (e.g., mentor role or level of formality). Future work is needed to better understand how specific characteristics of mentoring relationships, such as mentor role and level of formality, influence the degree of support students obtain. Future qualitative studies that examine differences in mentoring relationships (e.g., academic research mentors versus family or community members) will provide deeper insight into how these characteristics shape perceived mentoring support.

Another limitation arises in that our measurement of mentor-mentee similarity did not include psychological similarity, which has been shown to be a predictor of perceived mentoring support [17,58]. Students with a higher psychological similarity to their mentors may experience higher quality interactions and greater perceived support even with less frequent interactions. Although our analysis is consistent with previous work using experiential similarity as a proxy, future research should examine the role of psychological similarity in undergraduate BBS mentoring relationships.

In addition, although students were able to report multiple sources of mentorship, subsequent questions about mentoring characteristics (e.g., interaction frequency, relationship length, and perceived support) required students to respond with a single mentor in mind. As a result, it is unclear which mentor students were referencing when answering these items, particularly for those with multiple mentoring relationships. Students were asked to report on the mentor that had most impacted their career development, which could influence the observed patterns in mentoring support and relationship characteristics. Future research should consider designs that allow students to report on multiple mentors individually to better capture the complexity of their mentoring networks.

Finally, scalar invariance for the CSMS subscales could not be achieved, limiting our cross-institutional comparisons of mean differences. Together, these limitations emphasize the need for mentoring-focused survey instruments that demonstrate validity across institutions and support meaningful comparative evaluation of mentoring initiatives. Future research should integrate richer institutional contextual data and continue refining mentoring measurement tools to better capture how structural conditions shape mentoring experiences across institutional settings.

## Conclusion

Given the plethora of benefits that mentoring can provide to undergraduate BBS students [3–5], it is crucial to better understand the mentoring relationships of these students. In this study, we provided insight on BBS student mentoring at two large, public institutions. We observed that most BBS students reported having a mentor, with many of these mentors interacting with their mentees as peers or outside their institution. We also observed institution-specific patterns in mentor-mentee experiential similarity, interaction frequency, and relationship length. Although we provided evidence for validity for two scales of mentoring support, *Degree and Career Support* and *Existence of a Role Model*, we were unable to achieve full measurement invariance and compare mentoring support between institutions. Our correlational data provided a starting point to develop evidence-based recommendations for practice, summarized in Table 3. However, in order to compare mentoring outcomes across institutions, future research should focus on the development of survey instruments that can be applied broadly.

## Supporting information

S1 File S1-S5 Tables and captions, S1-S3 Figures and captions. (PDF)

S2 File S6 Table and caption. (XLSX)
