# Supplementary material for: Mentorship across institutional contexts: Insights from biology and biomedical science undergraduates at two institutions
Source: PLoS One. 2026 Aug 19;21(8):e0355290. doi: 10.1371/journal.pone.0355290 (PMC13489457; doi:10.1371/journal.pone.0355290)
Supplement: Supplementary file 1 [file pone.0355290.s001.pdf]

Supplementary materials

Table S1. Non-Likert scale items used in the mentoring questionnaire. The type of question and variable measured are listed, along with selectable options for multiple-choice questions.

Variable	Questions and Choices
**We define a mentor as a person who supports, advises, and guides you.**	
Source of Mentor (Yes/No, for each item)	Do you currently have a mentor at [your institution]?
	Do you currently have a mentor outside of [your institution]?
	Do you currently have more than one mentor?
	Do you currently have peer mentors?
	Have you been involved with an organized mentoring program?
**Reflect on one mentor in your life that you have learned the most from or has made a meaningful impact on your career development.**	
Experiential Similarity (Yes/No)	Is your mentor in the same career field you are pursuing?
Interaction Frequency	**How often do you meet with this mentor? (Choices below)**
	Daily
	2–3 times weekly
	Weekly
	2–3 times a month
	Monthly
	Less than once a month
Relationship Length	**How long have you been working with this mentor? (Choices below)**
	Less than one a month
	2–6 months
	6 months to 1 year
	1–2 years
	2–3 years
	3–4 years
	4 or more years

Table S2. Items used for both of the CSMS subscales measured. The means and standard deviations for each institution are listed.

Factor	Item Code	I have a mentor in my life who...	Inst. A		Inst. B	
			Mean	SD	Mean	SD
Degree and Career Support	DCS1	Encourages me to consider educational opportunities beyond my current plans	5.10	1.11	4.84	1.19
	DCS2	Helps me realistically examine my degree or certificate options	5.09	1.11	4.85	1.20
	DCS3	Questions my assumptions by guiding me through a realistic appraisal of my skills	5.04	1.13	4.79	1.14
	DCS4	Helps me carefully examine my degree or certificate options	4.98	1.18	4.70	1.18
Existence of a Role Model	ERM1	Shares personal examples of difficulties he or she has had to overcome to accomplish academic goals	5.14	1.10	4.93	1.10
	ERM2	Serves as a model for how to be successful in college	5.12	1.10	4.97	1.04
	ERM3	Sets a good example about how to relate to other people	5.18	1.06	5.03	1.06
	ERM4	I want to copy their behaviors as they relate to college-going	4.90	1.23	4.75	1.23
	ERM5	I look up to regarding college-related issues	4.99	1.20	4.87	1.20
	ERM6	I admire	5.36	0.96	5.20	0.96

Table S3. Model fit statistics for initial and revised confirmatory factor models of mentoring supports across institutional contexts.

	Institutional Context	χ^2 (df) *p*-value	CFI	RMSEA [90% CI]	SRMR
Initial Model	**Institution A** (n = 725)	$\chi^2(34) = 136.87$ $p < 0.001$	0.959	0.101 [0.083–0.119]	0.032
	**Institution B** (n = 746)	$\chi^2(34) = 106.75$ $p < 0.001$	0.973	0.080 [0.063–0.098]	0.036
Final Model	**Institution A**	$\chi^2(18) = 33.11$ $p < 0.001$	0.993	0.052 [0.020–0.081]	0.016
	**Institution B**	$\chi^2(18) = 28.19$ $p < 0.001$	0.996	0.039 [0.00–0.066]	0.015

Table S4. Standardized factor loadings from two-factor CFAs and reliability coefficients (ω_t = McDonald's omega reliability coefficient) for the survey by institution.

**Factors**	**Item Code**	**Institution A**	**Institution B**
Degree and Career Support	DCS1	0.803	0.775
	DCS2	0.905	0.880
	DCS3	0.893`	0.862
	DCS4	0.864	0.876
	Reliability ($\boldsymbol{\omega_t}$)	0.92	0.90
Existence of a Role Model	ERM2	0.889	0.843
	ERM3	0.893	0.854
	ERM4	0.830	0.819
	ERM6	0.807	0.791
	Reliability ($\boldsymbol{\omega_t}$)	0.92	0.90

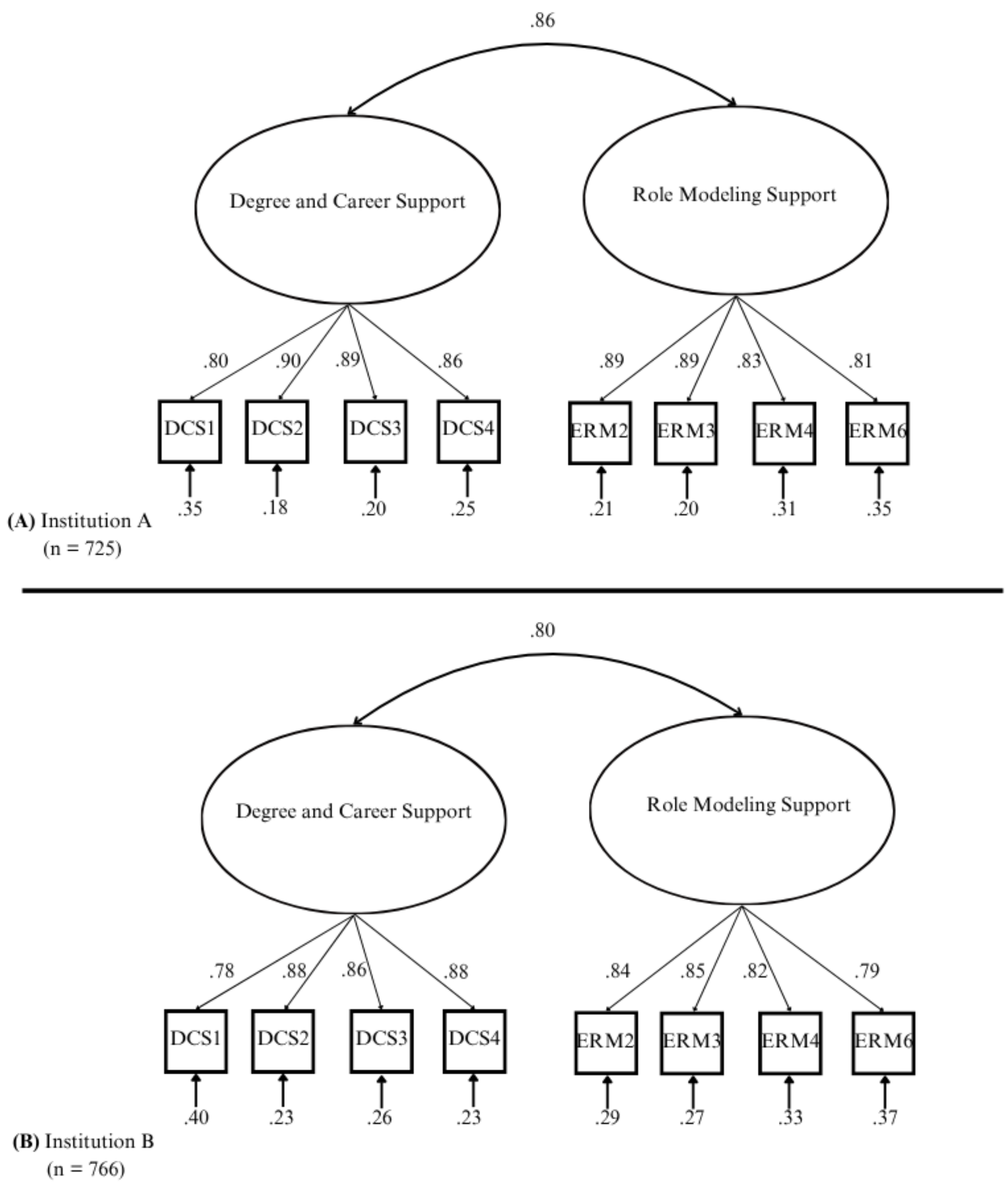

Fig S1. Two-factor models for (**A**) Institution A and (**B**) Institution B students who reported having at least one mentor. Instrument items (Table S2) are represented by the squares. The ovals represent the mentoring constructs from Nora and Crisp's (2007) model. *DCS* refers to *Degree and Career Support* and *ERM* refers to *Existence of a Role Model*. The numbers associated with the double headed arrows are the correlations between each factor. The numbers next to the unidirectional arrows that point toward the item squares are the standardized factor loadings. Small arrows below each item square indicate the error terms. $p < 0.000$ for all estimates.

Table S5. Measurement invariance tests on CFA models.

	**Institution A (n = 725) vs. Institution B (n = 766)**		
**Fit Statistics of Tests**	**Configural**	**Metric**	**Scalar**
Δχ2 value (p - value)	186.112 (< 0.001)	148.513 (< 0.001)	(< 0.001)
RMSEA [90% CI]	0.075 [0.064–0.086]	0.072 [0.063–0.081]	0.035 [0.027–0.044]
CFI	0.988	0.987	0.998
SRMR	0.015	0.017	0.016
**Comparisons of Tests**	**N/A**	**vs. Configural**	**vs. Metric**
Δχ2 value (p - value)	-	122.3 (< 0.001)	541.7 (< 0.001)
ΔRMSEA	-	−0.003	0.023
ΔCFI	-	−0.001	0.001
ΔSRMR	-	0.002	−0.002

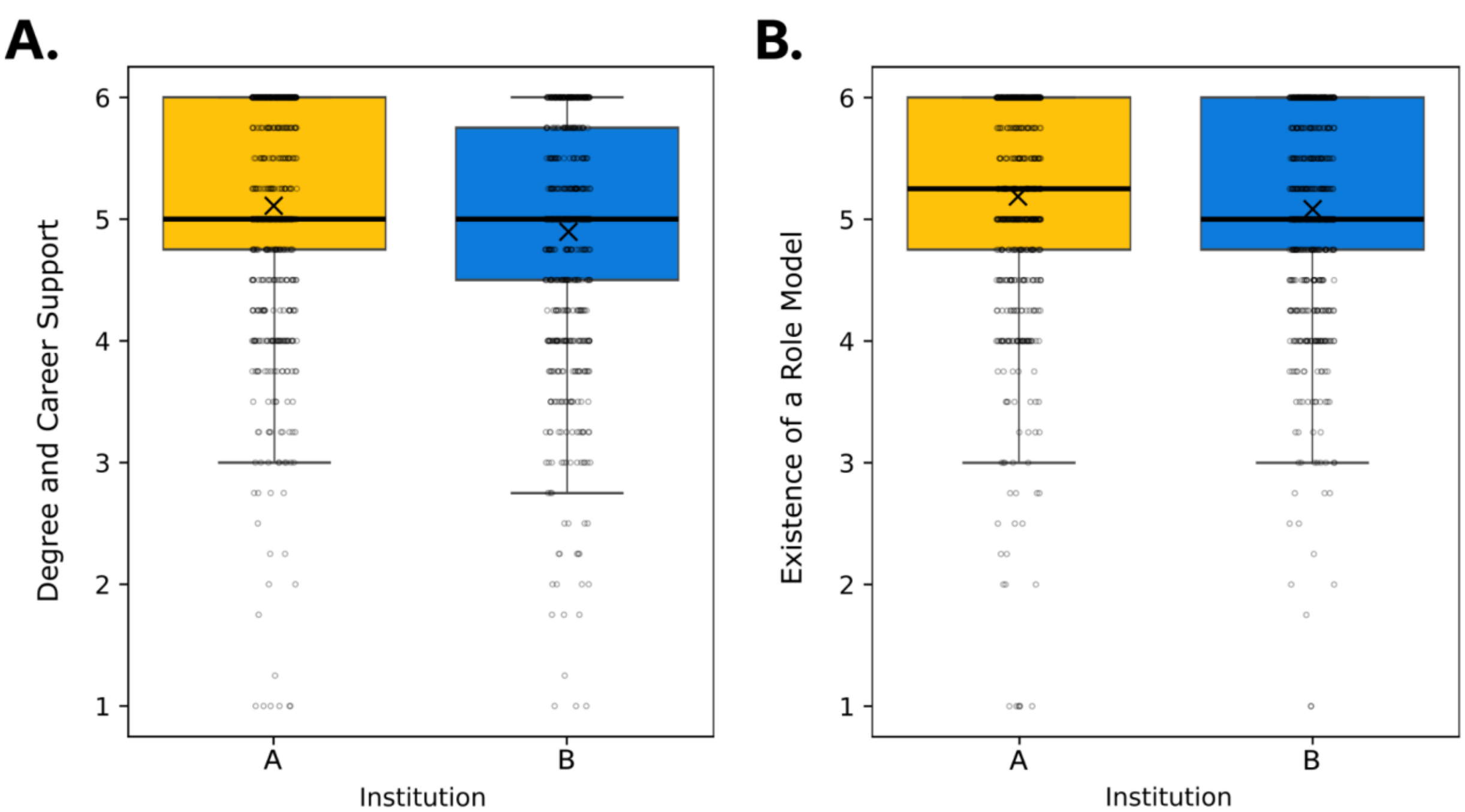

Fig S2. Distribution of mentoring support scales by institution. **(A)** *Degree and Career Support*, and **(B)** *Existence of a Role Model*. Institution A is in yellow (left box), and B is in blue (right box).

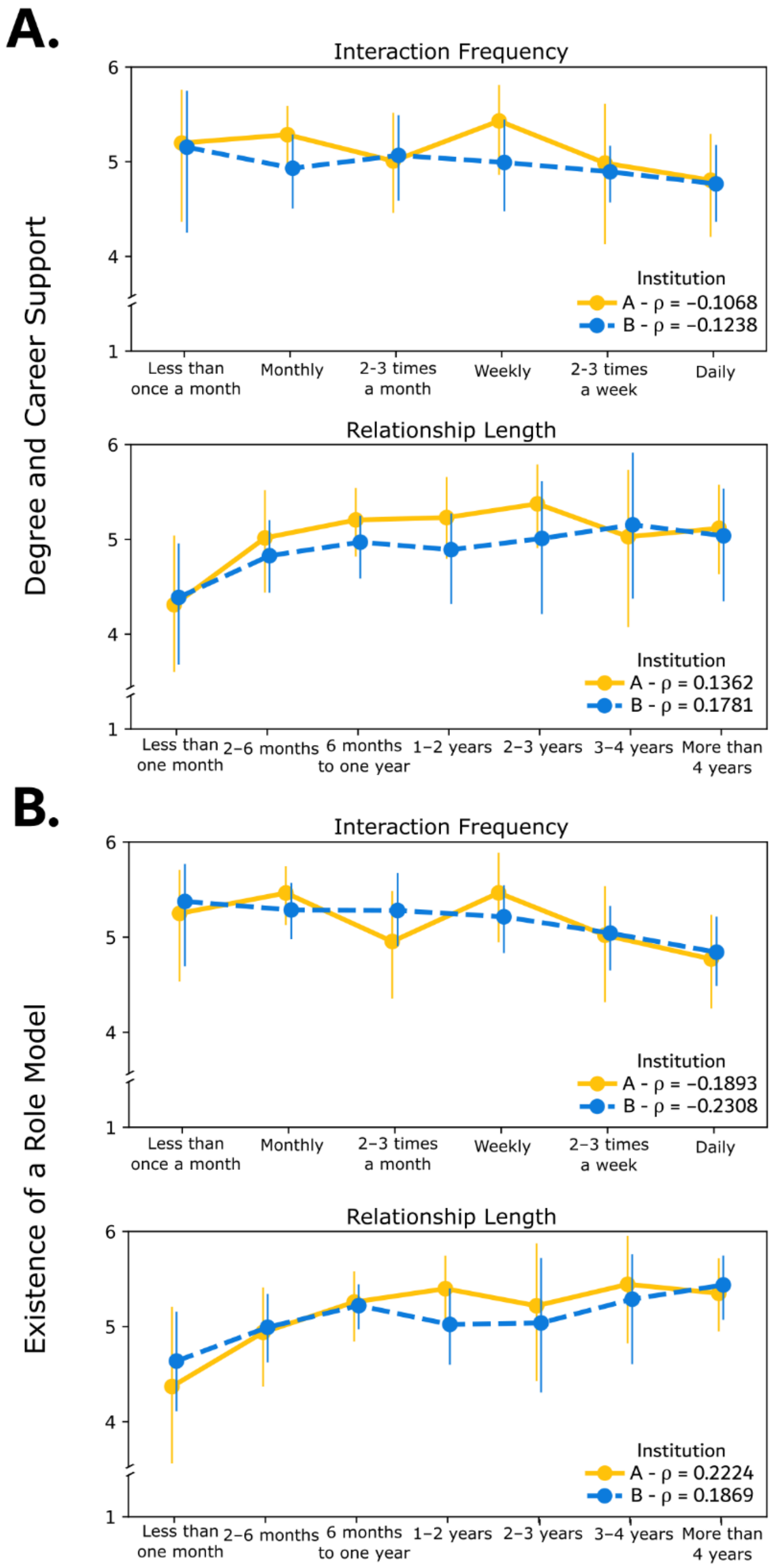

Fig S3. Mentoring support scale responses by mentor-mentee interaction frequency and relationship length only for students who reported mentors at their institution (A – n = 235, B – n = 310). **(A)** *Degree and Career Support*, **(B)** *Existence of a Role Mode*l. Institution A results are indicated by a yellow, solid line, while Institution B results are indicated by a blue, dashed line. Spearman's rho [ρ] values are indicated for each correlation.
